# Listening to Foreign Languages: Pump Up the Volume!

**DOI:** 10.5334/joc.445

**Published:** 2025-04-23

**Authors:** Boris New, Clément Guichet, Elsa Spinelli, Julien Barra

**Affiliations:** 1 Univ. Grenoble Alpes, France; 2 Univ. Savoie Mont Blanc, CNRS, LPNC, 38000 Grenoble, France

**Keywords:** spoken word recognition, word superiority effect, lexical feedback activation, TRACE model, cross-linguistic

## Abstract

In this study, we investigated whether the visual “word height superiority illusion” (New et al., 2016) could be found in the auditory modality. In two experiments, participants listened to a word–word or word–pseudoword pair of the same or different intensity and judged whether one was louder than the other. They judged stimuli from their native language (L1) and second language (L2). In Experiment 1 with native French speakers, we found that words were perceived louder than pseudowords in the L1 (French) and the L2 (English). Moreover, the illusion was stronger in the L1 (French) than in the L2 (English). In Experiment 2 with native English speakers, we replicated the illusion both in the L1 (English) and the L2 (French) but to a similar extent. Overall, we replicated the visual word height superiority illusion in the auditory modality, which suggests that this may reflect a more general cognitive mechanism.

## Introduction

A well-known effect in psycholinguistics is the *word superiority effect* (Reicher, 1969). This refers to how participants, when presented with a visual stimulus for a relatively short time and asked to identify the letter at a given position among two alternatives, tend to make fewer errors when the letter is presented within a word than within a pseudoword or in isolation. This lexical influence on letter perception is often interpreted within the framework of the interactive activation model (IAM; McClelland & Rumelhart, 1981). The IAM advocates for the following three types of processing units: those that code for features, those that code for letters, and those that code for words. When a stimulus is presented, activation flows up from the feature level to the letter and word level. Facilitatory feedback is then sent back from the word level down to the lower levels. Thus, a letter presented in a word receives both bottom-up and top-down activation from the feature and word level, respectively. By contrast, a letter presented in isolation (or in a pseudoword) only receives bottom-up activation. At a lower level of processing, the letter superiority effect (Reingold & Jolicoeur, 1993; Schendel & Shaw, 1976) suggests that a feature is better perceived in a letter than in a pseudoletter. This is explained by a similar feedback activation, but this time from the letter to the feature level. In the original IAM model, there are no backpropagation links from the letter level to the feature level, but this letter superiority effect reasonably suggests that there are.

Based on this framework, New et al. (2016) demonstrated the perceptual consequences of the word and letter superiority effects. They provided evidence for a new illusion whereby letters are perceived as taller than pseudoletters despite having the same objective height. This illusion also extended to words, which were considered taller than pseudowords made of pseudoletters of the same objective size and taller than pseudowords made of reversed syllables (see also Reber et al., 2004).

New et al. (2016) attempted to explain the illusion of letter height superiority through two mechanisms. First, the misperception of letter height is the perceptual consequence of the increased activation of features in letters (which is impossible with pseudoletters). Recall that in the IAM model, when a letter is presented, its features receive bottom-up but also top-down activation from the letter level. By contrast, when a pseudoletter is presented, its features only receive bottom-up activation. The perceptual consequence of the increased activation of features in letters, giving rise to the height illusion, comes from feedback from higher levels of processing (lexical level to letter level to feature level). Second, studies have demonstrated that the more active a feature is, the larger it is perceived: Barra et al. (2020) presented on a white background gray stimuli (a low signal-to-noise ratio) or black stimuli (a high signal-to-noise ratio). Participants judged the size of pairs of either letters or pseudoletters presented as black or gray. The results indicated that for identical objective sizes, participants perceived black stimuli to be taller than gray stimuli. Thus, the higher the signal-to-noise ratio, the taller the letter was perceived to be. Interpreted in the framework of the IAM model, the features of a letter have been activated more when presented in a black letter than in a gray letter. This supported the hypothesis that during the presentation of a stimulus, the more a feature is activated, the taller the stimulus (including that feature) is perceived to be.

The perceptual fluency hypothesis could also explain this illusion. This hypothesis suggests that stimuli processed more quickly are easier to recognize, preferred, or perceived as more frequent (Reber & Zupanek, 2002; Wänke, Schwarz, & Bless, 1995). Perceptual fluency affects physical properties such as clarity (Goldinger, Kleider, & Shelley, 1999), figure-ground contrast, or stimulus duration (Reber et al., 2004). Within this framework, letters would appear taller than pseudoletters because they are processed more fluently (Reber et al., 2004). However, explanations based on associative models (computational) and fluency (phenomenological) are not mutually exclusive. Backpropagation might be the mechanism underlying perceptual fluency.

In the present study, we sought to understand whether this illusion is specific to the written modality or whether it also impacts the spoken modality. This illusion could also be specific to language or reflect a more general mechanism involving feedback from higher to lower processing levels. Indeed, some effects found in language processing are modality-specific, such that brain areas seem dedicated to visual language processing (Dehaene & Cohen, 2011). For instance, the visual word form area (VWFA) is an area of the left occipito-temporal sulcus that is crucial when reading a word but not when listening to it. Lesions in this area often lead to alexia, a deficit in written word recognition, whereas other language abilities remain intact, such as auditory word recognition. Thus, we expected that if the illusion is a perceptual bias restricted to the VWFA, then it should not be found in the auditory modality.

An example of an effect that can be generalized independently of modality is the word superiority effect, originally found in the visual modality, has been extended to the auditory modality. That is, a phoneme is identified more quickly when presented in a word than in a pseudoword (Frauenfelder et al., 1990). In a similar fashion to the IAM, the TRACE model of spoken word recognition (McClelland & Elman, 1986) also captures the influence of lexicality on phonemic perception. In TRACE, phonemic perception is the product of dynamic top-down and bottom-up connections (Samuel, 1981). Indeed, a phoneme is more accurately identified if it is embedded in a word (Samuel, 2001) because it receives knowledge-driven activation at the phonemic level along with signal-driven activation at the lower acoustic level. More specifically, when an isolated word is heard, activation flows up to the lexical unit and activates the phonemic stage through feedback activation. Therefore, an ambiguous phoneme (e.g., on a continuum /d/-/t/) is more likely to be perceived as a /t/ if participants are presented with the word *task* against the pseudoword *dask* because it is coherent with the phonetic categorization of a known word (see the *Ganong effect*; Ganong, 1980). In the same way as for the IAM model, it is reasonable to think that in the TRACE model there are backpropagation links from the phoneme to the feature level.

The aim of this study was twofold: First, we aimed to investigate whether the letter height superiority illusion could be extended to the auditory modality. Because this illusion suggests that words are perceived as taller than pseudowords, we tested whether words could be perceived as louder than pseudowords. We chose to manipulate the loudness of the stimuli because this dimension intuitively corresponds to the size increase used in the visual modality. If a strong activation of visual features leads to an overestimation of size, it could be that a strong activation of auditory features should also lead to an overestimation of the loudness of sounds. Therefore, we hypothesized that a word would be perceived louder than a pseudoword despite the same objective intensity. We presented words (e.g., *lapin* /lapɛ̃/ “*rabbit*”) and their matched pseudowords consisting of the swapped syllables (e.g., *pinla* /pɛ̃la/), which is a condition that was tested in the visual modality by New et al. (2016).

Second, we aimed to investigate whether this auditory illusion could be modulated by the amount of lexical feedback available to the listeners. An interesting approach to modulating lexical feedback is to test the illusion in the second language (L2) modality. Language-specific cues are highly dependent on the language experience of the listener (Cutler, 2000, Samuel & Frost, 2015). In that aspect, L1 and L2 listeners seem to share “basic bottom-up acoustic processing” but do differ in “top-down lexical processing.” In a phonemic restoration experiment, Ishida and Arai (2016) found that native speakers restored missing phonemes more in words than in pseudowords, while non-native speakers restored missing phonemes in words and in pseudowords to a similar extent. These results suggest that top-down lexical cues are adopted differently by native and non-native speakers. Presumably, native listeners rely more on top-down processes due to more extensive lexical representations (see also Samuel & Frost, 2015). Thus, we expected the potential auditory illusion to be stronger when participants listened to their L1 than when they listened to their L2.

## Experiment 1 (Native French Speakers)

### Method

#### Participants

Seventy-seven participants (34 women and 43 men, *M* = 24.5 years old) were recruited through the Prolific^1^ interface (Palan & Schitter, 2018). They were paid *£2.25* to participate for approximately 18 minutes. All participants reported having no auditory impairment, were native French speakers, and had English as their L2. The experiment was hosted online on Prolific and created with the online study builder Lab.js (Henninger et al., 2021). The results were collected with OpenLab (Shevchenko, 2022).

#### Stimuli

The stimuli in each language were 16 words (eight French and eight English; see Appendix) and their 16 syllable-swapped matched pseudowords. These 16 French and English words were pretested on 32 native French speakers (15 women and 17 men, *M* = 25.9 years) through the Prolific interface and selected from among 60 words. All words had correct recognition above 80% (on average, 100% for French and 93.4% for English stimuli). The words were all bisyllabic and followed a CVCV phonological structure. According to the lexical database Lexique (Version 3.83; New et al., 2004), their subtitle lemma frequency ranged from 43.68 to 605.75 occurrences per million words in French and from 33.8 to 554.49 occurrences per million words in English. We ensured that the French and English words’ lexical frequencies were comparable (*t*(14) = 0.12, *p* = .90; French: *M* = 204, [*SD*] = 171; English: *M* = 193, *SD* = 167). We also ensured that their duration matched (*t*(14) = 1.5, *p* = .15; French: *M* = 554 ms, *SD* = 62 ms; English: *M* = 600 ms, *SD* = 60 ms). The pseudowords were created from the original words by swapping the syllables of the words (e.g., the word *bateau* ‘boat’ /bato/ was transformed into /toba/). For our pseudowords, we chose to swap the syllables rather than play them backward, since playing a stimulus backward would considerably distort the signal and its perceived loudness (Stecker & Hafter, 2000).

Independent syllables (e.g., /ba/ and /to/) were generated using the Mac OS Text to Speech synthesizer software and then pasted together using the SoX toolbox (version 14.4.2). Swapping syllables allowed us to control acoustic variables, such as syllable frequency and syllable duration, between the word and its matched pseudoword. For each of the 16 stimuli, two different intensities were created using SoX, with the louder one always 4 dB higher than the quieter one. The threshold was set at 4 dB because it was important to ensure that the difference between the stimuli was not too obvious but induced a certain level of uncertainty. Indeed, a difference that would be too large would have led to success in identical pair trials, thus preventing the detection of the illusion. Similarly, a smaller difference would have made the task too difficult and therefore irrelevant. Within each intensity condition, the intensity of all stimuli was normalized.

#### Procedure & Design

Before beginning the experiment, participants filled out a questionnaire that asked for details on their language background such as their father’s and mother’s language, their subjective level of verbal expression and comprehension in their L2 on a 4-point scale (beginner, intermediate, advanced or near-native) and their weekly usage of their L1 and L2 as a percentage.

After a quick test based on dichotic pitch to ensure that they were using headphones (Milne et al., 2021), they calibrated the sound level of their device to the lowest level required to hear a beeping noise (~350 Hz). They proceeded to the training session with three words for each language (which were not repeated later in the experiment) until they reached 60% of correct responses (see Appendix). A 60% accuracy criterion is almost twice the chance level. The task was difficult to maintain a certain level of ambiguity in the identical condition so that the illusion can be measured. Indeed, if the difference in sound intensity were greater, making the task easier, the identical pairs would be judged as identical despite the illusion.

The training consisted of presenting the same word twice, with different or identical intensities and a feedback on the correctness of the answer was given after the subject’s response. Each pair of words was presented 3 times, resulting in 9 training trials.

A trial began with the presentation of a central fixation cross for 200 ms, followed by the auditory presentation of the first stimulus. The second stimulus was presented 300 ms after the end of the first one. Finally, a white screen was presented until the participant responded. One of the two stimuli was always a word, while the other one could be either a word or a pseudoword. Participants compared the loudness of the stimuli within a pair and judged whether one of the two was louder. If they judged the two stimuli to be identical, they were instructed to press the down arrow on the keyboard with their dominant hand. By contrast, if they judged one stimulus to be louder than the other, they were instructed to press the arrow that denoted the position of the *louder* stimulus within the pair – that is, either the left or right arrow if this stimulus was the first or second one within the pair, respectively. The next trial began 750 ms after the participant responded. The experimental session in each language comprised 72 trials. In one-third of the trials (i.e., in 24 trials), the stimuli were of the same intensity (=) and the remaining items were of a different intensity (< or >). All words and pseudowords were presented in nine experimental conditions: word = word; word < word; word > word; word = pseudoword; word > pseudoword; word < pseudoword; pseudoword = word; pseudoword > word; and pseudoword < word. The experiment lasted approximately 18 minutes.

The experimental design had the following three within-subject factors: (a) *language proficiency* with two levels (L1 and L2); (b) *relative intensity* within the pairs with three levels (identical, stimulus 1 louder, and stimulus 2 louder); and (c) *pair type* with two levels (word–word and word–pseudoword). Two blocks of stimuli were used for L1 and L2. The order of the blocks was counterbalanced across participants, while the order of pair presentation within a block was randomized. Participants were offered a short break halfway through each block.

### Results

Seventy-one participants met the inclusion criterion of 60% correct responses when a word–word pair was presented in the same objective intensity condition. As a result, the data of six participants was not considered in the analyses. In L1 (French) and L2 (English), 15% and 17% of participants, respectively, did not make any mistakes. All 71 participants’ data were considered for inferential statistical analyses, but only the participants who made at least one mistake were retained to compute the distribution of errors. A sensitivity analysis in G*Power 3.1 (*Faul et al., 2007**) indicated the lowest effect size of the suffering manipulation we can detect given our design would be d = 0.34 (with 71 participants, 0.05 error alpha rate, 0.80 power, two tails)*.

The analyses were restricted to the word–pseudoword pairs of identical intensity. Indeed, in the condition where the stimuli are of different intensities, the difference in intensity was set subjectively. The results obtained could be quite different for other intensities. Consequently, only the identical intensity condition can be used to test our hypothesis for generalizability.

The auditory illusion was assessed by computing the direction of errors: word perceived louder or pseudoword perceived louder. Observations with a studentized deleted residual (*sdr*) ± 4 were considered for exclusion according to Judd et al.’s (2018) recommendations for outlier detection. On this basis, no other participants were excluded. The statistical analyses in our study were performed to assess whether words were consistently perceived as louder than pseudowords when presented at the same objective intensity. The key dependent variable was the proportion of errors, categorized by whether participants judged the word or the pseudoword as louder. Planned comparisons were conducted using paired-sample t-tests to determine if errors consistently favored words across conditions. Additionally, ANOVAs were used to assess the main effects and interactions of language (L1 vs. L2), stimulus type (word vs. pseudoword), and relative intensity.

French speakers made 9.3% of errors on L1 word–word trials and 22.4% of errors on L1 word–pseudoword trials. Among the 22.4% of errors, the planned comparison revealed that the word was more often judged as being louder: 70.2% vs. 29.8%, *t*(70) = 6.35, *p* < .001, *d* = 0.75, and 95% confidence interval (CI) [0.53, Inf].

**Figure 1:**
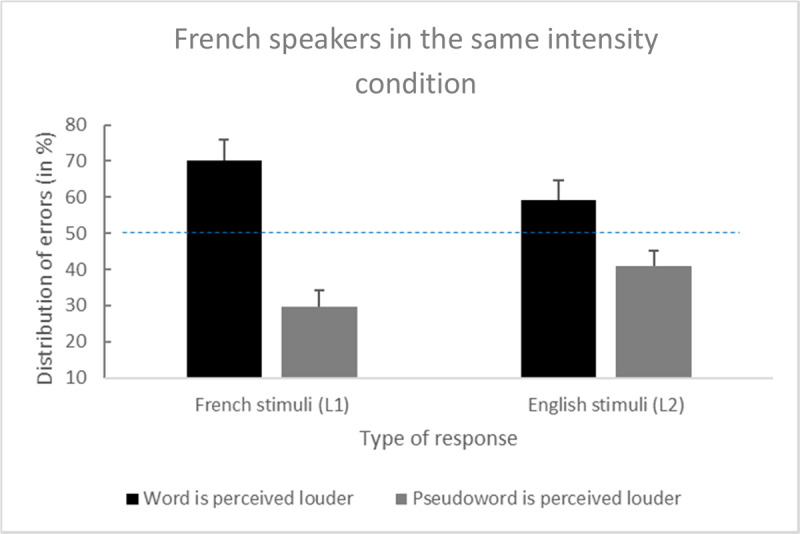
Distribution of errors among French speakers for the French (L1) and English (L2) stimuli (error bars are standard errors).

Moreover, participants made 7.7% of errors on L2 word–word trials and 20.5% of errors on L2 word–pseudoword trials. Among the 20.5% of errors, the planned comparison also revealed that the word was more often judged as being louder: 59.2% vs. 40.8%, *t*(70) = 2.4, *p* = .02, *d* = 0.28, and 95% CI [0.09, Inf] (see Figure 1).

The main effect of language was nonsignificant: F(1, 70) = .95, *p* = .33, η^2^_*p*_ = .01, and 95% CI [0.00, 0.09]. A significant interaction occurred between the main factors on the percentage of errors (i.e., a word’s loudness was overestimated significantly more often with the French than the English stimuli): *F*(1, 70) = 7.00, *p* = .01, η^2^_*p*_ = .09, and 95% CI [0.01, 0.21].

Experiment 1 revealed that when native French speakers failed to hear that a word and a pseudoword had the same intensity, they more often misperceived the word as the louder stimulus. This illusion was found in both L1 French and extended to L2 English. More interestingly, the illusion was stronger in L1 than L2 for native French listeners. This could be explained by less lexical feedback activation in L2 compared with L1. In Experiment 2, we investigated whether this illusion existed in English-speaking participants (i.e., participants with English as their L1 and French as their L2) using the same stimuli. Figure 1 presents Distribution of errors among French speakers.

## Experiment 2 (Native English Speakers)

### Method

#### Participants

Eighty-nine native English speakers (55 women and 34 men, *M* = 25.2 years) were recruited through the Prolific interface (Palan & Schitter, 2018). They were paid *£2.25* to participate for 18 minutes. All participants reported having no auditory impairment, were native English speakers, and had French as their L2. The experiment was hosted online on Prolific and created with Lab.js (Henninger et al., 2021). The results were collected with OpenLab.

#### Procedure & Design

We adopted the same procedure and design and used the same stimuli as described in the previous section for Experiment 1. It’s important to note that we used the same stimuli as we wanted to stick as closely as possible to the experiment with native French speakers, so we used the same stimuli composed of individually synthesized pronounced syllables. This means that, for words, stress is either absent or inappropriate. For French stimuli, stress should not play a major role since French is a syllable-time language and stress usually falls on the final syllable. On the contrary, English is considered as a stress-timed language explaining why stress is an important feature used to distinguish between words and meanings (Knight & Setter, 2021, Chapter 6, p. 159). The eight French and eight English words were pretested on 32 native English speakers (21 women and 11 men, *M* = 26.6 years) through Prolific. The results revealed that both the French and the English stimuli had above 80% correct recognition (on average, 88.7% for French and 91.4% for English stimuli). We can observe that the scores of English speakers are slightly lower than those of French speakers for stimuli in their native language. This result may be explained by the alteration of stress during stimulus composition.

### Results

Seventy-six participants met the inclusion criterion of 60% correct responses when a word–word pair was presented in the same objective intensity condition. As a result, the data of 13 participants were not considered in the analyses. In the L1 (English) and L2 (French), 4% and 8% of participants, respectively, did not make any mistakes. All 76 participants’ data were considered for inferential statistical analyses, but only the participants who made at least one mistake were retained to compute the distribution of errors. One outlier was excluded due to an *sdr* greater than 4 (*sdr* = 4.42). A sensitivity analysis in G*Power 3.1 (*Faul et al., 2007**) indicated the lowest effect size of the suffering manipulation we can detect given our design would be d = 0.33 (with 76 participants, 0.05 error alpha rate, 0.80 power, two tails)*.

English-speaking participants made 12% of errors on L1 word–word trials and 28.1% of errors on L1 word–pseudoword trials. Among the 28.1% of errors, the planned comparison revealed that the word was judged more often as louder: 58.1% vs. 41.9%, *t*(75) = 2.77, *p* < .01, *d* = 0.32, and 95% CI [0.12, Inf].

**Figure 2:**
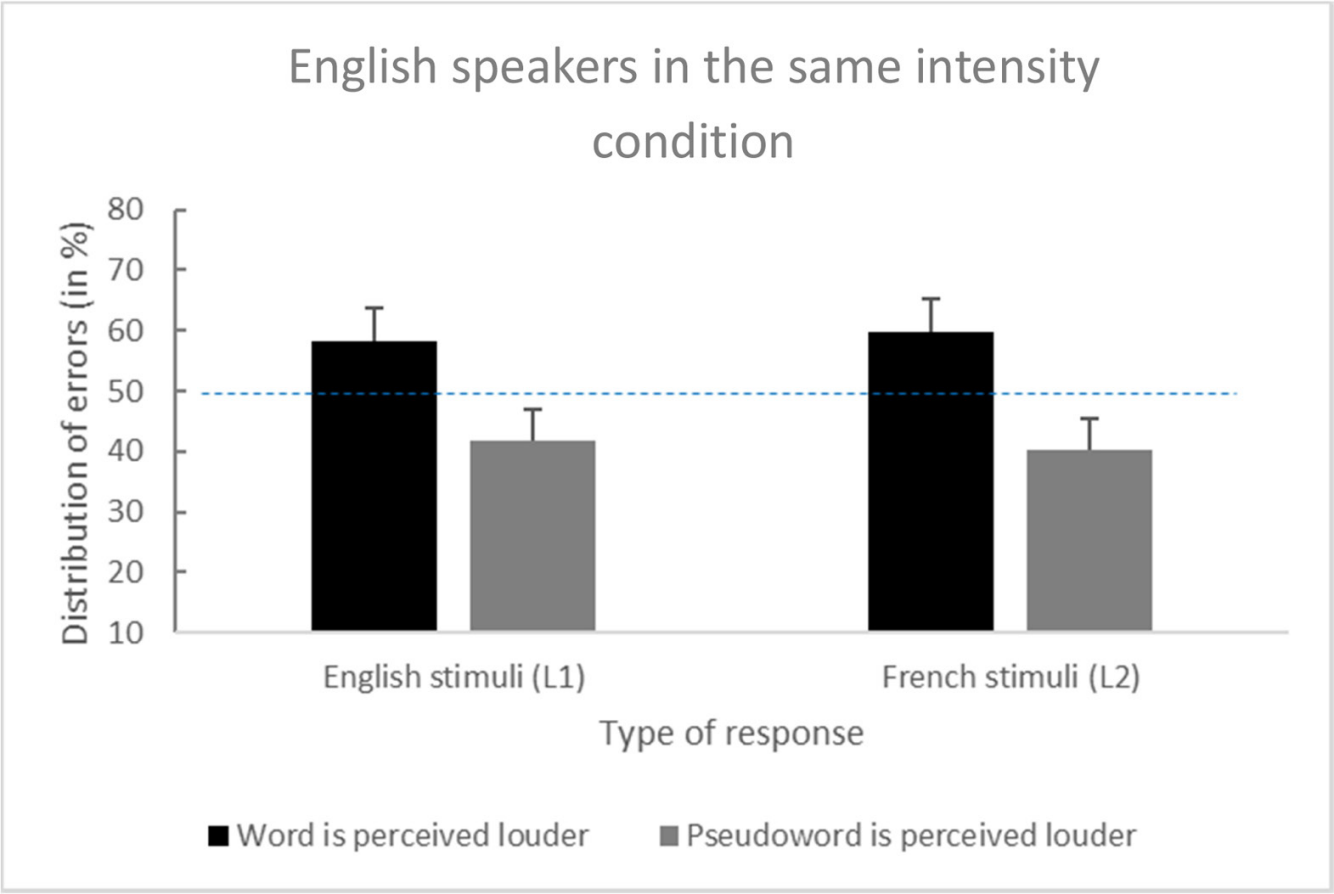
Distribution of errors among English speakers for the English (L1) and French (L2) stimuli (error bars are standard errors).

Moreover, the participants made 8.2% of errors on L2 word–word trials and 27.4% of errors on L2 word–pseudoword trials. Among the 27.4% of errors, the planned comparison also revealed that the word was judged more often as louder: 59.8% vs. 40.2%, *t*(75) = 3.48, *p* < .001, *d* = 0.40, 95% CI [0.20, Inf] (see Figure 2).

The main effect of *language* was nonsignificant: *F*(1, 75) = 0.07, *p* = .8, η^2^_*p*_ < .001, 95% CI [0.00, 0.04], which suggested that native English speakers did not make more perceptual errors in their L1 or L2. In contrast to native French speakers, the interaction between the main factors on the percentage of errors was nonsignificant: *F*(1, 75) = 0.17, *p* = .68, η^2^_*p*_ < .001, and 95% CI [0.00, 0.05]. Figure 2 presents the distribution of errors among English speakers.

In summary, native English speakers overestimated a word’s loudness when it was presented in a word–pseudoword pair of the same intensity in both their L1 and L2. However, the strength of the auditory illusion was comparable between the two languages.

## Participants characteristics analyses

Participants’ language proficiency was assessed through multiple measures. All participants declared their First Language (L1) and Fluent Language (L2) status on Prolific during recruitment. Additionally, they provided subjective ratings of their L2 verbal expression and comprehension abilities on a 4-point scale (beginner, intermediate, advanced, or near-native), as well as estimates of their weekly L1 and L2 usage percentages.

Statistical analyses of L2 proficiency ratings revealed significant violations of parametric test assumptions. The Shapiro-Wilk test indicated non-normal distributions for both production and listening conditions (p < .001), while Levene’s test showed significant heterogeneity of variances. Consequently, Mann-Whitney U tests were employed for group comparisons. French speakers reported significantly higher L2 proficiency than English speakers in both speaking (M = 3.15 vs. M = 2.64, U = 1819, p < .001) and listening skills (M = 3.42 vs. M = 2.76, U = 1591, p < .001).

Regarding weekly language usage, similar violations of normality and variance homogeneity were observed. Mann-Whitney tests revealed that English-speaking participants used their L2 (French) significantly less (M = 9.84%, SD = 14.2) than French-speaking participants used their L2 (English) (M = 32.4%, SD = 27.4), U = 1085, p < .001. Conversely, English speakers reported higher L1 usage (M = 87.41%, SD = 16.1) compared to French speakers (M = 68.2%, SD = 28.2), U = 1490, p < .001.

## Discussion

This study examined whether the word and letter height superiority illusion – which indicates that letters and words are perceived as taller than pseudoletters and pseudowords, respectively (New et al., 2016) – could be extended to the auditory modality. In two experiments, we found that words were perceived as louder than pseudowords. To our knowledge, this is the first demonstration of this illusion in the auditory modality. This illusion was proven to be robust as it was found every time we tested it (four times across our two experiments).

One possible explanation of this effect can be found within the framework of the TRACE model developed by McClelland and Elman (1986). This model uses an architecture similar to the IAM but applied to the auditory modality. It has three types of unit coding for phonetic features, phonemes, and words. Crucially, the TRACE model postulates both bottom-up and top-down activation. When a word is presented, it will activate word units that send excitatory feedback to the lower levels (phonemes and features). Although the TRACE model does not explicitly postulate backpropagation from phonemes to features, it is likely that this mechanism may be at work between these two levels too. In this case, when a pseudoword is presented, this will not activate word units, and therefore, less or no excitatory feedback will be sent to the lower levels (features).

An illustration of this excitatory feedback is the “word superiority effect,” which has been found in the auditory modality: in a phoneme monitoring task, a consonant is detected faster in a word than in a pseudoword, which suggests that lexical activation can influence phoneme perception. Moreover, having the target at the end of the stimulus when lexical activation is complete, instead of at the beginning, increases the probability of a word superiority effect being observed (Frauenfelder et al., 1990). Frauenfelder et al. (1990) asked their participants to detect a consonant (e.g., /p/) in words and pseudowords at various positions: at the beginning (e.g., /p/ in *pagina*, word vs. *pafime*, pseudoword), before the uniqueness point (e.g., /p/ in *operatie* vs. *opelakoe*), after the uniqueness point (e.g., /p/ in *olympiade* vs. *arimpiako*), and at the end of the word (e.g., /p/ in *bioscoop* vs. *deoftoop*). The word superiority effect was stronger after the uniqueness point, which indicated that phoneme detection can be influenced by lexical knowledge and lexical activation. In the framework of the TRACE model, this suggests that phonemes and possibly features are more activated in words, which is in contrast to the case in pseudowords. Here, the following question arises: What could the perceptive consequences be of such an increased activation of phonemes or features in words compared with pseudowords?

Neuroimaging studies on auditory intensity coding have demonstrated that neural activation increases in auditory areas with the sound pressure level. More specifically, most functional magnetic resonance imaging (fMRI) studies on the coding of sound intensity have found an increase in the activated cortex volume with sound intensity (Brechmann et al., 2002; Hall et al., 2001; Hart et al., 2003; Jäncke et al., 1998; Lasota et al., 2003; Röhl & Uppenkamp, 2012). Furthermore, most fMRI studies have found an increase in the BOLD signal strength with increasing sound intensity (see, e.g., Brechmann et al., 2002; Hall et al., 2001). In addition, the BOLD response in the auditory cortex seems to be linked to loudness rather than objective sound pressure (Röhl & Uppenkamp, 2012). Adopting the opposite reasoning, one can hypothesize that the stronger the activation of a representation in the auditory cortex, the louder the corresponding sound is perceived. Thus, if words activate phonemes or feature units more strongly than pseudowords do, then words would be perceived as louder. In another study, a similar reasoning was applied in the visual modality to explain why participants perceived a black stimulus (with a higher signal-to-noise ratio) as taller than a gray one (Barra et al., 2020). Correspondingly, in a different research field, Linkenauger et al. (2009) demonstrated that right-handed participants perceived their right arm and hand to be longer than their left arm and hand. Noteworthily, this misperception was also associated with a greater area in the left hemisphere responsible for the cortical representation of the right arm or hand. This suggests that a cortical representation may be related to the (mis)perception of one’s body. A similar mechanism may have been at play in the present study, whereby lexical representations are more likely to induce auditory misperceptions because of larger cortical representations. Note that such top-down mechanisms have been invoked to explain why, at an equal level of blur, predictable objects or scenes are perceived as sharper than unpredictable ones. Therefore, just as in this study, what makes sense in the environment helps people to perceive it more accurately (Rossel, Peyrin, Roux-Sibilon & Kauffmann, 2022). These two examples are consistent with the fact that our illusion is generalizable to known/familiar objects and not specific to language.

We investigated the existence of this new auditory illusion in French and English as an L1 or an L2 and evaluated the role of lexical feedback by assessing the differential strength of the illusion between the two languages. Taken together, the results of Experiments 1 and 2 support the idea that words are perceived louder than pseudowords, even in the L2 of the listener. Moreover, the results of Experiment 1 indicated that French listeners experienced a stronger illusion in L1 than in their L2 (i.e., English). This ties in with studies that have indicated that native listeners are better equipped to effectively combine bottom-up acoustic cues with top-down lexical processes (e.g., Ishida & Arai, 2016). Indeed, listeners are at a disadvantage when processing L2 stimuli because they do not benefit from strong top-down lexical activation. This is mainly due to them having a smaller mental lexicon in L2 as well as increased lexical competition (Broersma, 2012; Weber & Cutler, 2004). Recent neuroimaging studies have also provided evidence for this interaction; that is, non-native listeners exhibit cortical activity to a mostly syllabic acoustic rhythm, whereas native listeners’ cortical activity aligns with the rhythm of lexical structures (Ding et al., 2016; Wagner et al., 2022). Thus, one may conclude that the auditory illusion is dependent on the listener’s lexical knowledge.

Although we replicated the illusion with English listeners, such an interaction was not found in Experiment 2 with native English listeners. That is, the illusion was not stronger in L1 English than in L2 French, as expected. We argue that this may have been caused by the way we generated our stimuli. To keep a maximum number of parameters constant in our word–pseudoword pairs, we recorded individual syllables that we concatenated. Research has suggested that native English listeners rely on suprasegmental information (e.g., frequency and intensity) along with segmental information to signal lexical stress (Cho et al., 2007; Connell et al., 2018). More specifically, compared with native French listeners, native English listeners exhibit no trace of syllabic segmentation when listening to English or French words (Cutler et al., 1986). Given that native English listeners use their knowledge of stress patterns to decrease lexical competition, generating independent syllables and pasting them together may alter the stress pattern of the word (Cutler & Norris, 1988; Frost et al., 2017). In this regard, one could speculate that native English listeners may benefit from less feedback activation from the word units, which could exhibit a weaker auditory illusion in L1. By contrast, native French listeners were likely not disturbed by such alterations in L2 English because of their inability to encode contrastive stress in their phonological representations (i.e., a ‘**pre**sent vs. to pre’**sent**; Dupoux et al., 2001, 2008). Another possible explanation could be that this difference between French and English speakers stems from differences in their proficiency in their L2. Indeed, the analyses show both that French speakers seem to have a better mastery of their L2 than English speakers and that French speakers practice their L2 more frequently than English speakers. These differences could explain why English speakers might experience a weaker illusion in their L2 compared to French speakers, or why French speakers might experience a weaker illusion in their L1 compared to English speakers, however, this is not what we observe. On the contrary, we observe that English speakers have a weaker illusion than French speakers in their L1 (58.1% of words perceived as louder in a word-pseudoword pair for English speakers vs. 70.2% for French speakers) and a similar illusion in their L2 (59.2% of words perceived as louder in a word-pseudoword pair for English speakers vs. 59.8% for French speakers).

### Study limitations

People have individual differences in their sensitivity to loudness. To account for these variations in intensity detection and the different listening conditions, we have first asked participants to adjust the sound intensity to the lowest level at which they can detect a neutral stimulus. This procedure allows us to adapt stimulus intensity to inter-individual differences but is based on self-adjustment. This method has limitations and does not account for fine-grained hearing differences. In future studies, an audiometric test could be used as a pre-screening method to take fine-grained hearing differences into account.

Furthermore, the task used in the experiments was relatively demanding in terms of attention and concentration, which led to a limitation of the number of words per language to 8 in order not to exceed 20 minutes of experiment duration. In future studies, it would be interesting to use different words or a larger number of words to ensure that the results are independent of the stimuli used in our experiments and to draw more robust conclusions about linguistic and structural differences.

To further investigate the cross-linguistic robustness of the effect it would be valuable to replicate the experiment in another syllable-timed language such as Italian. Given that Italian, like French, is a syllable-timed language, one could expect Italian participants to exhibit a similar illusion to French participants if the stimuli were constructed using the same method.

## Conclusion

In summary, we demonstrated that the phenomena underlying the word height superiority effect also applied in the auditory modality. We demonstrated that stimuli that are highly activated (words) generate percepts (louder) that have no acoustical correlates. This also indicates that this illusion is not specific to the visual modality but may reflect a more general language or perceptual mechanism that is modulated by the listener’s linguistic experience. More specifically, we suggest that this illusion is stronger in the L1 than in the L2, thus underpinning the role that top-down lexical processes play in spoken word recognition. This may explain what some people have already experienced in their lives – namely that when they watch a movie in a foreign language, they tend to turn up the volume to understand the dialogues better.

## Data Accessibility Statement

Both of our experiments were preregistered: https://osf.io/qd26w (Experiment 1) and https://osf.io/ksw39 (Experiment 2). All of our analysis scripts and data can be found on our OSF page: https://osf.io/8kvma/?view_only=944a361f59984c618109411ccdd55836.

## Footnotes

https://www.prolific.co/.

## Appendix

**Table:**

LANGUAGE	WORD	ENGLISH TRANSLATION	PHONOLOGICAL TRANSCRIPTION	SWAPPED PHONOLOGICAL TRANSCRIPTION

French	café	coffee	/ka.fe/	/fe.ka/

French	chanter	sing	/ʃɑ̃.te/	/te.ʃɑ̃/

French	chateau	castle	/ʃɑ.to/	/to.ʃɑ/

French	chéri	honey	/ʃe.ʁi/	/ʁi.ʃe/

French	content	happy	/kɔ̃.tɑ̃/	/tɑ̃.kɔ̃/

French	maison	house	/mɛ.zɔ̃/	/zɔ̃.mɛ/

French	photo	photo	/fo.to/	/to.fo/

French	santé	health	/sɑ̃.te/	/te.sɑ̃/

English	coffee		/ˈkɒ.fi/	/fi.ˈkɒ/

English	father		/ˈfɑː.ðər/	/ðər.ˈfɑː/

English	forget		/fəˈ.ɡet/	/ɡet.fəˈ/

English	hero		/ˈhɪə.rəʊ/	/rəʊ.ˈhɪə/

English	major		/ˈmeɪ.dʒər/	/dʒər.ˈmeɪ/

English	safer		/ˈseɪ.fər/	/fər.ˈseɪ/

English	water		/ˈwɔː.tər/	/tər.ˈwɔː/

English	yellow		/ˈje.ləʊ/	/ləʊ.ˈje/

## References

[1] Barra, J., Pallier, C., & New, B. (2020). The black superiority effect: Black is taller than gray. Acta Psychologica, 202, 102958. 10.1016/j.actpsy.2019.102958 31864215

[2] Brechmann, A., Baumgart, F., & Scheich, H. (2002). Sound-level-dependent representation of frequency modulations in human auditory cortex: a low-noise fMRI study. Journal of Neurophysiology, 87(1), 423–433. 10.1152/jn.00187.2001 11784760

[3] Broersma, M. (2012). Increased lexical activation and reduced competition in second-language listening. Language and cognitive processes, 27(7–8), 1205–1224. 10.1080/01690965.2012.660170

[4] Cho, T., McQueen, J. M., & Cox, E. A. (2007). Prosodically driven phonetic detail in speech processing: The case of domain-initial strengthening in English. Journal of Phonetics, 35(2), 210–243. 10.1016/j.wocn.2006.03.003

[5] Connell, K., Hüls, S., Martínez-García, M. T., Qin, Z., Shin, S., Yan, H., & Tremblay, A. (2018). English learners’ use of segmental and suprasegmental cues to stress in lexical access: An Eye-Tracking study. Language Learning, 68(3), 635–668. 10.1111/lang.12288

[6] Cutler, A. (2000). Listening to a second language through the ears of a first. Interpreting, 5(1), 1–23. 10.1075/intp.5.1.02cut

[7] Cutler, A., Mehler, J., Norris, D., & Segui, J. (1986). The syllable’s differing role in the segmentation of French and English. Journal of Memory and Language, 25(4), 385–400. 10.1016/0749-596X(86)90033-1

[8] Cutler, A., & Norris, D. (1988). The role of strong syllables in segmentation for lexical access. Journal of Experimental Psychology: Human perception and performance, 14(1), 113. 10.1037/0096-1523.14.1.113

[9] Dehaene, S., & Cohen, L. (2011). The unique role of the visual word form area in reading. Trends in Cognitive Sciences, 15(6), 254–262. 10.1016/j.tics.2011.04.003 21592844

[10] Ding, N., Melloni, L., Zhang, H., Tian, X., & Poeppel, D. (2016). Cortical tracking of hierarchical linguistic structures in connected speech. Nature neuroscience, 19(1), 158–164. 10.1038/nn.4186 26642090 PMC4809195

[11] Dupoux, E., Peperkamp, S., & Sebastián-Gallés, N. (2001). A robust method to study stress “deafness”. The Journal of the Acoustical Society of America, 110(3), 1606–1618. 10.1121/1.1380437 11572370

[12] Dupoux, E., Sebastián-Gallés, N., Navarrete, E., & Peperkamp, S. (2008). Persistent stress ‘deafness’: The case of French learners of Spanish. Cognition, 106(2), 682–706. 10.1016/j.cognition.2007.04.001 17592731

[13] Faul, F., Erdfelder, E., Lang, A.-G., & Buchner, A. (2007). G*power 3: A flexible statistical power analysis program for the social, behavioral, and biomedical sciences. Behavior Research Methods, 39(2), 175–191. 10.3758/BF03193146 17695343

[14] Frauenfelder, U. H., Segui, J., & Dijkstra, T. (1990). Lexical effects in phonemic processing: Facilitatory or inhibitory? Journal of Experimental Psychology: Human Perception and Performance, 16(1), 77. 10.1037/0096-1523.16.1.77 2137525

[15] Frost, R. L., Monaghan, P., & Tatsumi, T. (2017). Domain-general mechanisms for speech segmentation: The role of duration information in language learning. Journal of Experimental Psychology: Human Perception and Performance, 43(3), 466. 10.1037/xhp0000325 27893268 PMC5327892

[16] Ganong, W. F. (1980). Phonetic categorization in auditory word perception. Journal of Experimental Psychology: Human Perception and Performance, 6(1), 110. 10.1037//0096-1523.6.1.110 6444985

[17] Goldinger, S. D., Kleider, H. M., & Shelley, E. (1999). The marriage of perception and memory: Creating two-way illusions with words and voices. Memory & cognition, 27, 328–338. 10.3758/bf03211416 10226442

[18] Hall, D. A., Haggard, M. P., Summerfield, A. Q., Akeroyd, M. A., Palmer, A. R., & Bowtell, R. W. (2001). Functional magnetic resonance imaging measurements of sound-level encoding in the absence of background scanner noise. The Journal of the Acoustical Society of America, 109(4), 1559–1570. 10.1121/1.1345697 11325127

[19] Hart, H. C., Hall, D. A., & Palmer, A. R. (2003). The sound-level-dependent growth in the extent of fMRI activation in Heschl’s gyrus is different for low-and high-frequency tones. Hearing research, 179(1–2), 104–112. 10.1016/S0378-5955(03)00100-X 12742243

[20] Henninger, F., Shevchenko, Y., Mertens, U. K., Kieslich, P. J., & Hilbig, B. E. (2021). lab. js: A free, open, online study builder. Behavior Research Methods, 1–18. 10.3758/s13428-019-01283-5 PMC9046347 34322854

[21] Ishida, M., & Arai, T. (2016). Missing phonemes are perceptually restored but differently by native and non-native listeners. SpringerPlus, 5(1), 1–10. 10.1186/s40064-016-2479-8 27375982 PMC4908083

[22] Jäncke, L., Shah, N. J., Posse, S., Grosse-Ryuken, M., & Müller-Gärtner, H. W. (1998). Intensity coding of auditory stimuli: an fMRI study. Neuropsychologia, 36(9), 875–883. 10.1016/S0028-3932(98)00019-0 9740361

[23] Judd, C. M., McClelland, G. H., Ryan, C. S., Muller, D., & Yzerbyt, V. (2018). Analyse des données: une approche par comparaison de modèles. De Boeck Superieur.

[24] Knight, R. A., & Setter, J. (Eds.). (2021). The Cambridge handbook of phonetics (pp. 159–184). Cambridge University Press. 10.1017/9781108644198

[25] Lasota, K. J., Ulmer, J. L., Firszt, J. B., Biswal, B. B., Daniels, D. L., & Prost, R. W. (2003). Intensity-dependent activation of the primary auditory cortex in functional magnetic resonance imaging. Journal of computer-assisted tomography, 27(2), 213–218. 10.1097/00004728-200303000-00018 12703014

[26] Linkenauger, S. A., Witt, J. K., Stefanucci, J. K., Bakdash, J. Z., & Proffitt, D. R. (2009). The effects of handedness and reachability on perceived distance. Journal of Experimental Psychology: Human Perception and Performance, 35(6), 1649. 10.1037/a0016875 19968426 PMC3291021

[27] McClelland, J. L., & Elman, J. L. (1986). Interactive processes in speech perception: The TRACE model In Parallel Distributed Processing: Explorations in the Microstructure of Cognition, Vol. 2: Psychological and biological models (pp. 58–121).

[28] McClelland, J. L., & Rumelhart, D. E. (1981). An interactive activation model of context effects in letter perception: I. An account of basic findings. Psychological review, 88(5), 375. 10.1037//0033-295X.88.5.375 7058229

[29] Milne, A. E., Bianco, R., Poole, K. C., Zhao, S., Oxenham, A. J., Billig, A. J., & Chait, M. (2021). An online headphone screening test based on dichotic pitch. Behavior Research Methods, 53, 1551–1562. 10.3758/s13428-020-01514-0 33300103 PMC7725427

[30] New, B., Doré-Mazars, K., Cavézian, C., Pallier, C., & Barra, J. (2016). The letter height superiority illusion. Psychonomic bulletin & review, 23, 291–298. 10.3758/s13423-014-0753-8 26370216

[31] New, B., Pallier, C., Brysbaert, M., & Ferrand, L. (2004). Lexique 2: A new French lexical database. Behavior Research Methods, Instruments, & Computers, 36(3), 516–524. 10.3758/BF03195598 15641440

[32] Palan, S., & Schitter, C. (2018). Prolific. ac—A subject pool for online experiments. Journal of Behavioral and Experimental Finance, 17, 22–27. 10.1016/j.jbef.2017.12.004

[33] Reber, R., Zimmermann, T. D., & Wurtz, P. (2004). Judgments of duration, figure–ground contrast, and size for words and nonwords. Perception & Psychophysics, 66, 1105–1114. 10.3758/BF03196839 15751469

[34] Reber, R., & Zupanek, N. (2002). Effects of processing fluency on estimates of probability and frequency. In P. Sedlmeier & T. Betsch (Eds.), ETC. Frequency processing and cognition (pp. 175–188). Oxford University Press. 10.1093/acprof:oso/9780198508632.003.0011

[35] Reicher, G. M. (1969). Perceptual recognition as a function of meaningfulness of stimulus material. Journal of Experimental Psychology, 81(2), 275. 10.1037/h0027768 5811803

[36] Reingold, E. M., & Jolicoeur, P. (1993). Perceptual versus postperceptual mediation of visual context effects: Evidence from the letter-superiority effect. Perception & Psychophysics, 53(2), 166–178. 10.3758/BF03211727 8433915

[37] Röhl, M., & Uppenkamp, S. (2012). Neural coding of sound intensity and loudness in the human auditory system. Journal of the Association for Research in Otolaryngology, 13, 369–379. 10.1007/s10162-012-0315-6 22354617 PMC3346895

[38] Rossel, P., Peyrin, C., Roux-Sibilon, A., & Kauffmann, L. (2022). It makes sense, so I see it better! Contextual information about the visual environment increases its perceived sharpness. Journal of Experimental Psychology: Human Perception and Performance, 48(4), 331. 10.1037/xhp0000993 35130017

[39] Samuel, A. G. (1981). The role of bottom-up confirmation in the phonemic restoration illusion. Journal of Experimental Psychology: Human Perception and Performance, 7(5), 112. 10.1037/0096-1523.7.5.1124 6457110

[40] Samuel, A. G. (2001). Knowing a word affects the fundamental perception of the sounds within it. Psychological Science, 12(4), 348–351. 10.1111/1467-9280.00364 11476105

[41] Samuel, A. G., & Frost, R. (2015). Lexical support for phonetic perception during nonnative spoken word recognition. Psychonomic bulletin & review, 22, 1746–1752. 10.3758/s13423-015-0847-y 26866066 PMC4822410

[42] Schendel, J. D., & Shaw, P. (1976). A test of the generality of the word-context effect Perception & Psychophysics, 19, 383–393. 10.3758/BF03199397

[43] Shevchenko, Y. (2022). Open Lab: A web application for running and sharing online experiments. Behavior Research Methods, 54(6), 3118–3125. 10.3758/s13428-021-01776-2 35233751 PMC9729124

[44] Stecker, G. C., & Hafter, E. R. (2000). An effect of temporal asymmetry on loudness. The Journal of the Acoustical Society of America, 107(6), 3358–3368. 10.1121/1.429407 10875381

[45] Wagner, M., Ortiz-Mantilla, S., Rusiniak, M., Benasich, A. A., Shafer, V. L., & Steinschneider, M. (2022). Acoustic-level and language-specific processing of native and non-native phonological sequence onsets in the low gamma and theta-frequency bands. Scientific reports, 12(1), 314. 10.1038/s41598-021-03611-2 35013345 PMC8748887

[46] Wänke, M., Schwarz, N., & Bless, H. (1995). The availability heuristic revisited: Experienced ease of retrieval in mundane frequency estimates. Acta Psychologica, 89(1), 83–90. 10.1016/0001-6918(93)e0072-a

[47] Weber, A., & Cutler, A. (2004). Lexical competition in non-native spoken-word recognition. Journal of memory and language, 50(1), 1–25. 10.1016/S0749-596X(03)00105-0

